# Breaking the Spell: Combating Multidrug Resistant ‘Superbugs’

**DOI:** 10.3389/fmicb.2016.00174

**Published:** 2016-02-18

**Authors:** Shahper N. Khan, Asad U. Khan

**Affiliations:** Interdisciplinary Biotechnology Unit, Aligarh Muslim UniversityAligarh, India

**Keywords:** multidrug-resistant, drug development, functional genomics, network pharmacology, *in vivo* imaging

## Abstract

Multidrug-resistant (MDR) bacteria have become a severe threat to community wellbeing. Conventional antibiotics are getting progressively more ineffective as a consequence of resistance, making it imperative to realize improved antimicrobial options. In this review we emphasized the microorganisms primarily reported of being resistance, referred as ESKAPE pathogens (*Enterococcus faecium*, *Staphylococcus aureus*, *Klebsiella pneumoniae*, *Acinetobacter baumanii*, *Pseudomonas aeruginosa*, and *Enterobacteriaceae*) accentuating their capacity to “escape” from routine antimicrobial regimes. The upcoming antimicrobial agents showing great potential and can serve as alternative therapeutic options are discussed. We also provided succinct overview of two evolving technologies; specifically network pharmacology and functional genomics profiling. Furthermore, *In vivo* imaging techniques can provide novel targets and a real time tool for potential lead molecule assessment. The employment of such approaches at prelude of a drug development process, will enables more informed decisions on candidate drug selection and will maximize or predict therapeutic potential before clinical testing.

## Introduction

A change in the pattern of serious hospital infection after the introduction of antibiotics was noticed early in the antibiotic era. Furthermore with the triumph of the penicillin discovery and the synthesis of antibacterial sulfonamides in the initial half of the 20th century, the modern antimicrobial revolution started. There onward, newer antimicrobial were reported and several semi-synthetic and synthetic antibacterial agents came into existence. Antimicrobial drugs have cured plenty of cases with life-threatening bacterial infections and relieved patients’ agony. Unfortunately, unrestrained use of antibacterial past 50 years has wielded selection pressure on susceptible bacteria stains, which attributed to the endurance of drug resistance (Levy and Marshall, 2004; Tacconelli, 2009), among them some are resistant to more than one antibiotic. Presently, the treatment of these infections has once again becomes increasingly complicated as microorganisms are becoming resistance to the available antimicrobial options (Pitout and Laupland, 2008; Nordmann et al., 2011; Khan and Nordmann, 2012a; Labro and Bryskier, 2014). With course of time, sustained selective pressure by various antibiotics has culminated into organisms augmenting ancillary resistance mechanisms that led to multidrug resistance (MDR)—novel penicillin-binding proteins (PBPs), enzyme dependant drug alteration, altered membrane permeability, mutated drug targets and increased eﬄux pump expression. Further to mention few most challenging MDR organisms presently being encountered includes the so called ESKAPE pathogens like *Pseudomonas aeruginosa*, *Acinetobacter baumannii*, *Escherichia coli*, and *Klebsiella neumoniae* with extended-spectrum β-lactamases (ESBL), vancomycin resistant enterococci (VRE), methicillin-resistant *Staphylococcus aureus* (MRSA), vancomycin-resistant MRSA strains, extensively drug-resistant (XDR) *Mycobacterium tuberculosis* and newly identified transmissible carbapenamase, New Delhi metallo-beta-lactamases (NDM) in Enterobacteriaceae (Alekshun and Levy, 2007; Gootz, 2010; Khan and Nordmann, 2012b). Carbapenems were the only sensitive antibiotics for the treatment of MDR coliforms but the development of carbapenem resistance recently is a matter of great concern. Efforts directed toward identifying newer antibiotics were formerly an exquisite research area and development priority among pharmaceutical giants but poor success rate has dampens the interest. Further studies comprehending resistance illustrates that the evolution and spread of antimicrobial resistance (AMR) is in fact, a very convoluted issue. Hence, a sole target will not ensure eradication of AMR; rather a coordinated multidisciplinary approach is needed to tackle this problem (Smith et al., 2009; Cantas et al., 2013). Mortality rates and length of hospital stay associated with the treatment of drug resistant infections are about twice as big when patients infected with drug sensitive bacteria of the same species, thereby ensuing inflation in healthcare costs.

The aim of this article is to emphasize the ever growing problem of antimicrobial resistance, counting in the present approaches to limit the spread of MDR. We specifically highlighted how the emerging technologies could be a great promise for new antimicrobial discovery.

## Failure of Present Measures to Combat MDR

Multidrug-resistant is prevalent in nature, and the strategy of eliminating resistance genes makes no sense, as the natural function of most resistance genes is not primarily confirming MDR (Morar and Wright, 2010). Most probably, there is a huge “intrinsic resistome” in bacterial organisms, composed of various genes with diverse phylogeny which contribute to resistance only on interaction with the antibiotic (Fajardo et al., 2008; Girgis et al., 2009; Sommer et al., 2009). Possible strategy to combat with emerging drug resistance is to control the emergence, selection, and spread of MDR strains of bacteria from hospital settings and community (Wright, 2009). The conventional strategies of combating the emergence and spread of MDR atypically relies on the discovery of newer drugs (Wright, 2009; Nordmann et al., 2012), reduction in antibiotic induced bacterial mutation, genetics dependant recombination and horizontal-transfer at lower concentration of drugs (Couce and Blázquez, 2009), suppression of phenotypic traits of resistance (Udekwu et al., 2009), use of combinations therapy (De Cristóbal et al., 2008), including antagonistic drugs (Drew, 2009), early intensive (frontline) therapy, maintaining a low bacterial density (Motter, 2010), and lately, surveillance of hypermutable organisms (Oliver et al., 2000; Carattoli, 2009) and targeting regulating functions necessary for infection (Dandekar and Dandekar, 2010; Greenberg et al., 2010). In essence, these preventive measures are proving increasingly inadequate in the existing global scenario of MDR (Boucher et al., 2009). Averting the spread of resistance can apparently be significant for the person, but exhibits weak impact on the community (Durante-Mangoni and Zarrilli, 2011). Procedures that might work in the initial phase of the development of resistance in hospitals or nations with low rates of MDR, may not be competent enough in regions with prevailing higher resistance frequency (De Gelder et al., 2007). Reports from regions with low levels of MDR such as Sweden, revealed that discontinuance of trimethoprim use for 2 years had no impact on the resistance rate of *Escherichia coli*. That was further linked to the extensive dissemination of trimethoprim resistance genes (*dfr*) along with other resistance determinants, thus encouraging co-selection of *dfr* with other resistance genes as well (Brolund et al., 2010). In a global scenario, eventually, resistance commencing in these heavily contaminated “sources of resistance” will infest areas that are still cleaner (Thaller et al., 2010). Thus, antibiotic resistance keep evolving to attain higher antibiotic resistance exhibiting elevated bacterial genetic evolvability, a phenomenon known as “genetic capitalism”, where the rich tends to become richer. It refers to further adaptive potential of an organism to enrich their resistance mechanism either via mutational or gene acquisition events.

## Potential Therapeutic Interventions

### Molecules Targeting Protein Synthesis

**FIGURE 1:**
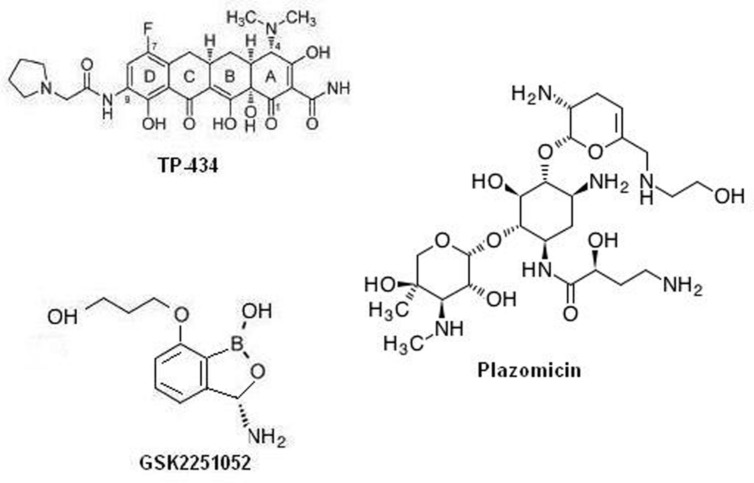
**Chemical structure of protein synthesis inhibitors as promising antimicrobial agents**.

Among the many exploited targets the one, which has been frequently approved by nature and by many successful clinical trial is the ribosome. Many antibiotics targeting protein synthesis are in development or in different phases of clinical trials as a prospective remedy of temperate-to-serious community acquired bacterial infections: solithromycin, cethromycin, omadacycline, CEM-102, GSK1322322, radezolid, and tedizolid. Interestingly, antibiotics targeting the cellular protein synthesis, TP-434, GSK2251052, and plazomicin, are reported to have a range that covers drug resistant gram negative class of bacteria. Among which, TP-434 (**Figure 1**) is a C7, C9 di-substituted broad-spectrum tetracycline based antibiotic was discovered and being developed by Tetraphase Pharmaceuticals, MA, showed effective mechanism-driven impediment of protein synthesis in a coupled transcription/translation assay [half maximal inhibitory concentration (IC_50_) = 0.29 μg/mL] and a competition assay employing radio labeled tetracycline (Grossman et al., 2012). Furthermore, TP-434 activity is also reported against tetracycline-specific eﬄux proteins (including *mepA*) genes bearing stains, ribosomal defense mechanisms and for enzymes capable of destructing tetracycline antibiotics (Sutcliffe et al., 2013). Further, TP-434 is insusceptible to *bla*_NDM-1_ positive carbapenemase that has recently been reported in enterobacteriaceae. Plazomicin (ACHN-490, **Figure 1**), a “neoglycoside,” a semisynthetic drug evolved from sisomicin with considerably enhanced efficacy toward amikacin and gentamicin resistant bacteria (Aggen et al., 2010; Armstrong and Miller, 2010). The minimum inhibitory concentration (MIC) _50/90_ reported for plazomicin ranges in 0.5–1 μg/mL against resistant *K. pneumoniae*, including the serine carbapenemase KPC strains and 1/2 μg/mL against 493 strains of MRSA positive bacteria (Tenover et al., 2011). Whereas, MICs tested against 65 carbapenem resistant enterobacteriaceae was found to be ≤2 μg/mL except for NDM metallo-β-lactamase producers (Livermore et al., 2011). Plazomicin has successfully concluded a level 2 in global multilocation indiscriminate double blind trial evaluating its efficacy relative to levofloxacin, a gold standard for complex urinary tract infection (cUTI) and acute pyelonephritis (AP) and now registered in a level 3 trial stage (ClinicalTrials.gov identifier: NCT01970371). GSK2251052 (**Figure 1**), is a novel boron derivative that interacts with the leucyl-tRNA synthetase at adenosine ribose (A76) termini with an IC_50_ of 0.31 μM, thereby aborting protein synthesis (Goldstein et al., 2013). The compound has shown variable range of MIC_50_*_/_*_90_ for various rampant gram negative aerobic and anaerobic bacteria and anaerobic gram-positive strains (Mendes et al., 2013). Further, in an *in vitro* potency test against bla_NDM-1_ producing enterobacteriaceae isolates, MIC_90_ of the compound was reported to be 1 μg/mL, with a range of 0.5–2 μg/mL. This novel antibiotic has also shown is activity toward category A and B bacterial biothreat infections, with MIC_90_ concentration in the rage of 0.12–2 μg/ml (Heine et al., 2011). When tested on healthy cohort as single IV doses of 200, 400, 900, 2,000, or 3,000 mg (1-hour infusion) and as multiple ascending doses (MADs) of 500, 750, 1,200, or 2,000 mg two times a day for 8 days (500 mg) or 14 days (all other cohorts) was well taken, with no severe implications leading to patients withdrawal but a recent article reports development of some resistance toward it due to specific mutations in the LeuRS editing domain (O’Dwyer et al., 2015). A review by Sutcliffe on antibiotics targeting protein synthesis describes them in detail (Sutcliffe, 2011). Specifically, Dewan et al. (2014) describes in detail the importance aminoacyl-tRNA synthetases as a therapeutic target for novel antibiotic development.

### Peptides

Combinational administration of antimicrobial peptides (AMP’s) with other antimicrobials has been recommended as an alternative strategy for enhanced therapeutic outcomes. They act by affecting cell membrane integrity, inhibiting protein and cell wall synthesis, altering enzyme activity, and others (Guilhelmelli et al., 2013). Their discrete therapeutic potency and less side effects makes AMP’s interesting candidates for concurrent or subsequent use in diverse bacterial infections. AMP’s are recently regarded as a viable option for eradicating the multidrug resistant micro-organisms, based on their distinct mechanism of action than that of the presently used antibiotics (Fjell et al., 2011; Nan et al., 2012). Till date, more than 3700 AMP’s have already been reported and characterized from flora, faunna, and other sources (Tossi and Sandri, 2002). AMP’s can also be produced synthetically and moreover, recombinant AMP’s can be yielded by various recombinant cell and/or organisms (Yu et al., 2010). A good number of synthetic AMPs are being tested in clinical trials and in excess of 15 peptides or mimetics are in (or have completed) clinical testing as antimicrobial agents. In a phase 1/2 of clinical testing, the AMP hLF1–11 (1–11 amino acids long amino-terminal of human lactoferrin) was found to be risk free with no after effects when delivered intravenously (Velden et al., 2009). Two peptides, pexignan and omiganan have exhibited efficacy in Phase III clinical trials but waiting for approval. Pexiganan (Lipsky et al., 2008), a derived from magainin, demonstrated similar effectiveness to an oral fluoroquinolone for treating patients with diabetes foot ulcer but was not approved by the US Food and Drug Administration, although there is indication that it might resurface in clinical trials. Omiganan (MBI226), a derivative of indolicidin, proved competent in drastically limiting catheter colonization and microbial tunnel infections during catheterization (ClinicalTrials.gov identifier: NCT00608959). AMP’s when tested for treating UTI, the most common infections in hospital settings and well documented in general population, seems to work best due to the optimum environment where peptides naturally, exhibit their potency (Lupetti et al., 2003; Melendez-Alafort et al., 2004). Cudic et al. (2003), illustrated that higher salt concentrations decreases the antimicrobial activity of various AMP’s, however, lactoferricins and derivates thereof, are quite active against UTI, regardless of limited antimicrobial activity at higher salt concentration as suggested in *in vitro* settings. Probably, the peptide is effective in eradicating urinary tract infections at remote sites, likely through passive or active transfer of the peptide to the contagion site via renal discharge (Del Olmo et al., 2009; Fornili et al., 2010).

Administration of antimicrobial peptides treated equipment offers potential microbial protection, although the attrition in antimicrobial attributes due to cross linking of the peptide to solid supports must be addressed (Onaizi and Leong, 2011). There are satisfying instances for cationic peptides possessing clinical efficacy (Gordon et al., 2005); polymyxin a cationic lipopeptide is the last resort treatment for multi-drug resistant *Pseudomonas* and *Acinetobacter* infections, the gramicidin S a cyclic cationic peptide is frequently prescribed in topical and eye infections, and the cationic nisin antibiotic is already an approved food supplement in Europe. As per our inference, the increasing accessibility and exploitation of high throughput involved strategies has shown substantial prospects to improve the search for the next generation therapeutic peptides and peptide mimetic as antimicrobials not only specific for bacteria that are resistant to existing antibiotics but also for eliminating other disease causing bugs like protozoa, helminthes, insects and fungi (Bommarius and Kalman, 2009; Tavares et al., 2013).

### Vaccines

Rational designing of vaccines have markedly improved with the advancement of recombinant DNA technology against complex human pathologies. In spite of extraordinary performances in pre-clinical and clinical testing, only few recombinant vaccines are deemed safe for humans use till date. Wherein, extensive screening procedures for its production and speculations about their use might be accountable. Recent application of new approaches like systems biology and advanced immunology to better understand mechanism of vaccines is propelling the evolution of vaccinology. Moreover, the outcomes of synthetic biology in vaccine design as evident in the first synthetic bacterium, demonstrates its distinction and rational attributes. Over the last few years there have been several excellent research articles describing strategies of creating the ‘optimal’ vaccine, as well as highlighting the significant challenges of developing an effective vaccines (Otto, 2010; Wu et al., 2012). Inactivated bacterial strains have shown to be extremely immunogenic against Gram-negative bacteria and exhibit protective immunity against several other bacterial infections. However, its applicability in human is limited due to the presence of lipopolysaccharide induced endotoxin levels. Nevertheless, in a recent report it was suggested that lipopolysaccharide impaired and inactivated *A. baumannii* whole cells can induce protection against *A. baumannii* infections. In which, the endotoxins levels were found to be reduced (<1.0 toxin unit/10^6^ cells) relative to the wild type strain, as on inactivation of lipopolysaccharide due to a mutation in the lpxD gene (García-Quintanilla et al., 2014). Vaccines, intended at combat *P. aeruginosa* infections in cystic fibrosis have been developed and are under different clinical phases, though none has been recommended for use (Johansen and Gøtzsche, 2015). Besides the ongoing improvement in whole-cell enterotoxigenic *Escherichia coli* (ETEC) vaccine development the research on subunit or polypeptide vaccines, offers great therapeutic promise. Recent article by Zhang and Sack (2015), has critically summarizes the progress in the development of ETEC vaccine.

Despite the daunting odds of success, there are at least seven active vaccine efforts are ongoing in different stages of clinical development (GlaxoSmithKline/Nabi, Pfizer/Inhibitex, Sanofi Pasteur/Syntiron, Novartis, Novadigm, Integrated Biotherapeutics, and Vaccine Research International). Several studies have been conducted to evaluate the potential of vaccination with microbial surface components illustrating adhesive matrix molecules (MSCRAMMs) whole protein or subunit offers superior protection against staphylococcal infection. Earlier reported, recombinant parts of collagen adhesin (CNA) were shown to provide immunity in a murine model of sepsis (Kuklin et al., 2006). Shkreta et al. (2004) illustrated the protective potential of both clumping factor A (ClfA) and Fibronectin binding protein (FnBP) as an optimal vaccination strategy in bovine mastitis model, eliciting cellular and humoral immune response providing partial protection against *Staphylococcal mastitis*. Recently in an *In silico* analysis, potential multisubunit vaccine targets like ClfA, ron-regulated surface determinant (IsdB) and gamma hemolysin (Hlg) were predicted against *S. aureus* (Delfani et al., 2015). Likewise, several extracellular and surface protein factors in combination with aluminum hydroxide have shown to induce high and broad protection against *S. aureus* (Bagnoli et al., 2015).

Another promising alternative to vaccine prophylaxis against bacterial pathogenesis is the ‘DNA-vaccine’, wherein DNA sequence encodes the antigen(s) against which an immune response is sought. It will be less labor intensive and may offer a unique method for vaccination (Tang et al., 1992). Though to obtain an improved immunogenic response of DNA vaccines entails detailed familiarity of underlying mechanism through which DNA vaccines provide immunity to the patient and needs the right combination adjuvants as crucial molecules for strengthening the response (Coban et al., 2008). In implant infections caused by *S. aureus*, MSCRAMM is reported to act as an excellent immunogen for an anti-*Staphylococcus* vaccine. A survey comprising multiple studies in where MSCRAMMs of staphylococcal are exploited as the immunogenic entity, which can implemented following the start of infection as presented in a recent review by Arciola et al., where the authors suggests a “Decalogue” for a rational selection of specific target in constructing DNA vaccines to impede infections in implants (Arciola et al., 2009). Another proposed strategy to induce protective immunity against infectious diseases is the application of live attenuated vaccines. Recent studes illustrates that nasopharyngeal colonization with *Streptococcus pneumoniae* induces protective immunity against subsequent invasive infection, suggesting live vaccination through attenuated bacteria could be a treatment of choice in future. However the bacterial attributes influencing the strength of this adaptive immune response are still to be explored (Cohen et al., 2012).

## The Search of Novel Antimicrobials

### Concept of Network Pharmacology

The conception of directed “magic bullets” has long been a therapeutic goal to achieve since Paul Ehrlich (Strebhardt and Ullrich, 2008) and a realistic standard in drug development for past three decades. The central concept of drug development is the notion of developing maximally selective molecules directed to discrete drug targets. Conversely, current therapeutics affects multiple proteins rather than discrete target to execute its effect. Development in the area of systems biology further indicates phenotypic robustness and a network structure implying that specific ligands, compared to multi-target ones, exhibit inferior to expected clinical potency. This revised paradigm about polypharmacology offers improved strategy to rise upon the major pitfalls in new antibiotic development, i.e., efficacy and toxicity. Combining system biology with polypharmacology brings in the opportunity of increasing the existing druggable targets. Nevertheless, the coherent design of polypharmacology needs to fulfill the requirement for new procedures to confirm target associations and augment structure-activity interactions while preserving drug like attributes. Progresses in similar avenues are paying the path toward the next paradigm in novel antibiotic discovery as ‘network pharmacology’.

The notion about ‘one drug for one target’ has affected various facets of drug development approach, like disease categorization, target validation, drug design, and planning of clinical testing. Though, a growing data in post-genomics research is suggesting a more complicated scenario in drug action. An interesting research by Yildirim et al. (2007), illustrates not only the existence of several keys for each lock but also a single key for multiple locks. Employing network analysis of combined profiles and a network distance metric, can differentiate between soothing drugs, which ease symptoms, and drugs affecting directly the concern disease genes. Furthermore, the trends in recent years toward the drugs that target the genes associated with disease were emphasized by the network distance metric. It has been well accepted that many effective drugs in therapeutic areas as varied as oncology, psychiatry and antimicrobials affects multiple rather than single targets (Paolini et al., 2006; Tian and Liu, 2012). Many efficient antibiotics are directed toward multiple proteins concurrently rather than individual protein (Lange et al., 2007). For instance, the antimicrobial property of beta-lactams is reliant on the inhibition of more than one PBPs. Indeed, as multiple PBPs can be eliminated with least effect on phenotype (Denome et al., 1999), the approach of lone target essentiality may not have revealed this significant group of antimicrobial drug targets. Likewise, fluoroquinolone based antibiotics can inhibit more than one target proteins ParC and GyrA (Janoir et al., 1996). D-Cycloserine is reported to inhibit four proteins, dimer of alanine racemases and D-Ala-D-Ala ligases. Similarly, fosfomycin circumvent the excess of UDP *N*-acetylglucosamine enolpyruvyl transferases by restraining them both. Consequently, any effort in the direction of drug designing for combating drug resistance, could consider the development of procedures to identify and evaluate which group of targets can be repressed by one single drug and are indispensible, either alone (‘dualessentials’) or when taken together (‘synthetic lethals’).

Network biology is suggested to play imperative role in drug target characterization. Is it feasible to recognize drug targets by tracing their location in a biological network? When the researchers mapped drug targets against human protein interaction data, result suggests that drug targets tend to have more interactions than average proteins but fewer interactions than crucial proteins to a statistically significant degree (Hopkins, 2007, 2008). The findings about the linkage among drug targets that are not exclusively essential, opens up the avenues for statistical analysis to serve as a vital tool for scoring them. Conventionally, medicinal chemists considered the design of ligands with multiple activities with apprehension, skeptical about conjugated ligands with high molecular weights complex and/or their structure-activity relationships (Hopkins et al., 2006; Morphy and Rankovic, 2006). Though, polypharmacology of standard drugs illustrates that a more opportunistic plan exploring multi-target action could be more efficient. Employing chemogenomics along with network biology will bring in a efficient network-pharmacology approach to drug design (Keiser et al., 2007; Morphy and Rankovic, 2007). Developing strategy to assist polypharmacology will further help to predict better efficacy and redundant off-target properties.

### Functional Genomics in Drug Designing

**FIGURE 2:**
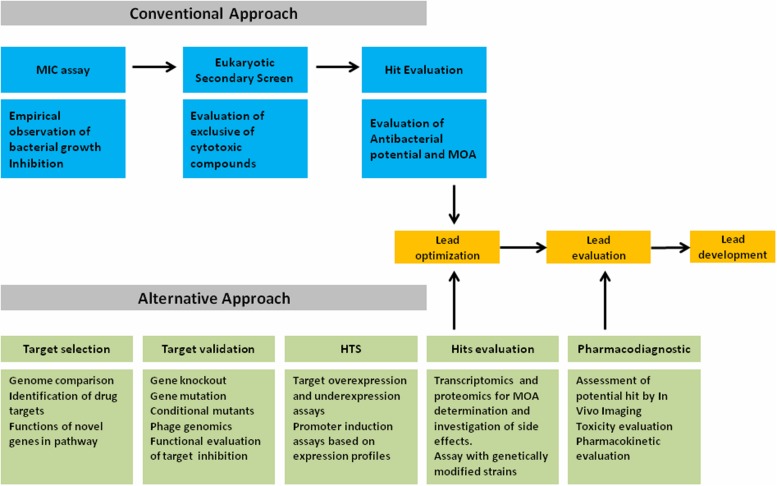
**Alternative approach for the discovery of next generation antibiotics**.

The tremendous advancement in the technology for genome screening had a substantial impact on all biological sciences, including antimicrobial research. Since the decoding of the *Haemophilus influenza* genome (Fleischmann et al., 1995), various other bacterial genomes were deduced. Complete genomes of >180 bacteria were sequenced and are now freely accessible, including various imperative disease causing pathogens (e.g., http://www.genomenewsnetwork.org/resources/sequenced_genomes/genome_guide_p1.shtml). Therefore, the drug development industry exploited the sequencing data as the basis for a rational, target-specific novel drug designing approach to complement the conventional strategy. Central to the paradigm shift was the idea of the occurrence of the so-far unexploited targets with the prospective for effective and discerning antibiotics for broad spectrum of bacterial infections (McDevitt et al., 2002; Gwynn et al., 2010; Brunschweiger and Hall, 2012). An array of genomics platforms are employed in the present antiinfectious drug development process to lay more proficient procedure compared to conventional strategy. Herein, we proposed a more robust strategy as an alternative for the discovery of next generation antibiotics (**Figure 2**), which includes all possible coverage for better output. Including knockout analyses and mutation studies in the strategy will enhance the prioritization and validation of prospective novel drug target by questioning its importance for bacterial existence. Applications of genetically manipulated cell, along with holistic profiling strategies like ‘OMICS,’ i.e., transcriptomic and proteomic are contributory in verifying the mode of action (MOA) while screening lead molecules from target assays. Functional genomics strategies also offers vital clues on the mechanism of lead molecules screened through the empiric approach for novel antibacterial discovery. Furthermore, comparing genomes facilitates the identification of proteins that are conserved across the clinically critical pathogens, assisting functional characterization of new genes and identification of novel targets. Genome wide gene silencing studies facilitate an approximation of the number of genes necessary for survival of various bacterial species. The inference on the role of same gene product essentiality (‘ortholog’) in different species will augment the feasibility of such targets. While selecting a target diverse criterion, such as screening competence, drug like attributes and metabolical context are of a great significance. Although, data on these features are often not accessible and even some targets are not completely characterized. The number of uncharacterized targets typically increases when focusing on attributes preserved in a selective group of pathogens (narrowed range of bacteria). Growth studies with different supplements, cytological assessment, transcriptome, proteome analyses, and metabolic labeling experiments of conditional mutants may assist in the evaluation of corresponding functional genes. Further interesting strategy to explore novel drug is to evaluate the molecular action of bacteriophages to arrest critical cellular pathways. As done by Liu et al. (2004), on sequencing 26 *S. aureus* phages thirty one (31) novel polypeptide were identified that on expression exhibit cell death in *S. aureus*. Affinity chromatography illustrates the role of DnaI as a target protein, a requisite protein for primosome activity on the initiation of DNA replication. Such screening techniques can be further extended to other species as well and could be instrumental in proving imperative information and as screening tools for novel or unexplored targets. In addition functional genomics applications have proved to be indispensable for expediting the characterization of mechanism of action for novel compounds with antinfectious activity and offers novel mechanism based screening options by generating and characterizing point mutations that confers drug resistance. In general conditional mutants with target gene repression illustrate an increase in sensitivity toward that target specific inhibitor. When analyzed, the relative growth suppression of these mutants to the wild type enables an easy validation confirming the mechanism of novel drugs/hits, as reported by DeVito et al. (2002) and Forsyth et al. (2002).

An approach of global expression profiling can revolutionize the analysis of drug targets holistically as never before. As whole genome profiling offers the static blueprint of cells attributes, as being highly dynamic in nature the transcriptome and proteome are appropriate to obtain snapshots of that cell physiology when challenged by environmental alterations, exposure to stress or antibiotic administration. As application of a DNA microarray provides direct assessment of the mRNA of the gene of interest, the proteomic profiling by 2D gel electrophoresis involves an implicit protein atlas needed initially to confirm the identity of each protein (as determined by mass spectrometry) to its spot location on the 2D gel (Brötz-Oesterhelt et al., 2005). The expression profiles resulted from these techniques illustrates the molecular insight of the adaptive physiology of bacteria like the regulatory mechanism, signal transduction and the underlying metabolism that influence the phenotypic outcomes. Transcriptome as well as proteome profiling were widely used to study the physiology of bacteria in response to various environmental stresses provided particularly useful information about the underlying network of adaptive responses. As a proof-of-concept is the *B. subtilis* mutant down regulating the peptide deformylase, wherein the proteomic profiling of this strain matched adequately well with the wild-type strain exposed to the deformylase inhibitor actinonin (Bandow et al., 2003). Furthermore, bacterial acetyl-CoA carboxylase (ACC) is recently been suggested as the target of moiramide B based on a reference compilation of transcriptome profiles (Freiberg et al., 2004, 2005). In a recent interesting work Murray et al. (2015) has employed expression- and fitness-based genomic strategies to validate role of genetic loci exhibiting drug resistance in human pathogen *P. aeruginosa*. Signifying that gene expression standalone is not a reliable predictor of resistance determinants. Moreover when combine together, whole genome expression and fitness correlation may offer better mechanistic perceptions about multidrug resistance.

### *In Vivo* Imaging Technologies

**FIGURE 3:**
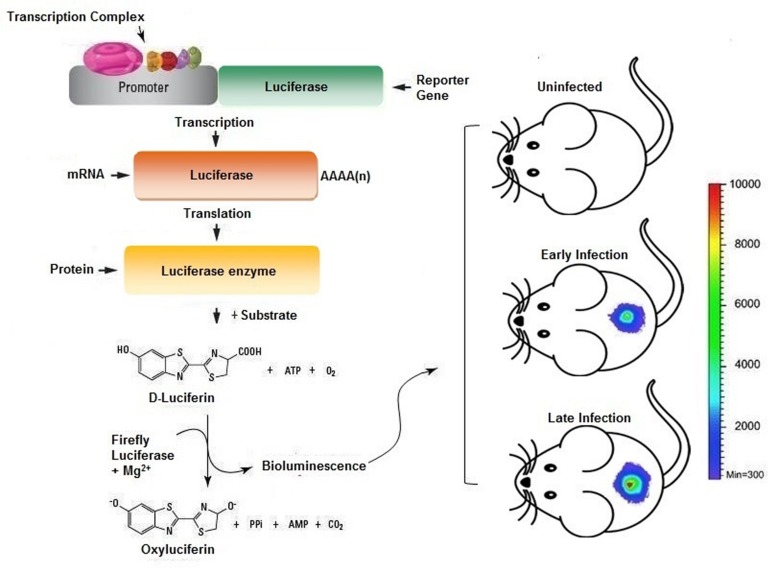
**Scheme representing the usage of *in vivo* imaging technique for the assessment of bacterial infection in murine model.** The bacterial cells bearing plasmid with reporter genes expressed will provide luminescence signals when injected with appropriate substrate provides real time readout for the extent of infection and/or therapeutic efficacy.

Recently, *in vivo* imaging techniques are being proved to be a powerful complement to various platforms of antimicrobial resistance and drug discovery (Probst et al., 2012; Roda and Guardigli, 2012). It may not only minimize the use of histopathological examination of autopsies but also be helpful in limiting the animal sacrifice and cost for *in vivo* drug-kinetic studies. Hence, we propose that improvement of intravital dynamic monitoring strategies to probe cellular phenotypes will reflect as wise drug development investment. Real time monitoring enables a faster readout of drug action in the complex model organisms, thus facilitating comprehensive assessment of drug profiling *in vivo*. Moreover, this could enable lead optimization and medicinal chemistry can be directed by instructive functional *in vivo* response data. The clinical reliability of highthroughput screens and *in vivo* monitoring strategies are yet to be fully comprehended. In particular, the progress in bioluminescence based monitoring of disease progression *in vivo* is ever increasing. Further identifies underlying physiology, such as signal transduction, proliferation and cell renewal to be examined in the purview of an intact organism. Two commonly employed strategies for incorporating bioluminescent marker in a model organism are: First is the *lux* operon from *Photorhabdus luminescens* or *Xenorhabdus luminescens*, encodes genes to produce luciferase and its substrate luciferin constitutively, and glow even absence of any exogenous substrate. The *lux* operon gets stably incorporated to other bacterial species but fail to integrate in higher species, though continuous efforts are make to use it as a reporter in mammalian system. The second strategy for bioluminescent probes uses enzyme luciferase from firefly (*Photinus pyralis*) or sea pansy (*Renilla reniliformis*), as firefly and Renilla luciferase require two distinct substrates, which make it possible to link them with two different biological processes in the same research model (Bhaumik and Gambhir, 2002; Ng et al., 2011). However, each reporter differs in the pharmacokinetic of their substrate, as on intraperitoneal administration of luciferin, firefly luciferase bioluminescence attain saturation in about 5–10 min and stays almost constant for 30 min (Paroo et al., 2004), however, renilla luciferase reach to its maxima in 1 min on intravenous coelenterazine administration and declines back in 10 min (Bhaumik and Gambhir, 2002). This technology can be successfully employed to study a variety of infection and their progression in animal models (**Figure 3**). Also manifested by the work of other researchers, that bioluminescence imaging can be used to validate new features in host–pathogen interactions. Furthermore developments in the live cell-fluorescent reporter molecules, brings in the opportunity to adopt *in vivo* monitoring applications in drug development (Carragher et al., 2012).

Past few years, array of nanoparticles have emerged with a promise of superior atttributes for *in vivo* fluorescence imaging technology. Besides providing dynamic size range, these particles offers diverse structural and optical properties with superior signals strength compared to organic fluorophores, providing more sensitive visualization of even sparse biomarkers. Furthermore, nanoparticles can incorporate multiple probes due larger surface area, facilitating multivalent targeting. Above mentioned attributes make these nanocarriers fit for therapeutic applications, biomedical imaging or a combination of both (theranostics). Quantum dots (QDs) are the best characterized and most studied nanocrystals (Mandal and Parvin, 2011; Li et al., 2012). These minute nanomaterials are semiconductive in nature and have an adjustable emission fluorescence that can be adjusted by regulating their synthesis (Bentolila et al., 2009). Furthermore can be visualized in a wide, from the ultraviolet to the near infrared exhibit higher intensity and photoresistant attributes. Recently, QDs treated with coordination complexes were used to specifically visualize a rough *Escherichia coli* mutant facilitating optical detection in murine infection model (Leevy et al., 2008). Various other nanoparticles that have been developed for *in vivo* fluorescence imaging applications are metallic, hybrid, C dots, polymeric or biodegradable in nature (Coto-García et al., 2011). Recent development in capturing detailed images and image based analysis softwares are beginning to impact the design of algorithms that can be customized to fit intricate and pertinent biological models and tissue specimen (Jones et al., 2009). Amendable image analyzing software’s can leverage quantitative data from complex biological systems, integrating varied cell types, fresh biopsies, or material from cell lines or primary cells for efficient mimicking of the cellular environment. Therefore, high-resolution *in vivo* imaging should facilitate drug discovery per se and understanding of the drug therapeutic effectiveness (**Table 1**), and will apparently increase the success rate in clinical testing by providing pertinent biological perspective to genomic and proteomic approaches.

**Table 1:** Potential applications of *in vivo* imaging in drug discovery.

Assessment of disease model efficiency	Disease model can be assessed for the optimal engraftment or infection from orthotropic cells before treatment regimes
Potential hit screening	Evaluation of MIC values across multiple pathways to determine the response of target activity
Biomarker discovery	Detection of post-translational markers of therapeutic relevance from clinical biopsy or body fluids
Identify drug–target mechanisms	Identification and validation of drug combination strategies and synergism of drugs
Predictive *in vivo* pharmacodynamics	Monitor organ-specific response and correlate with functional drug response
Drug candidate profiling *in vitro*	Establish broad pathway-activity mechanisms
Confirming functional genomics screens and pathways	Characterize impact of knockdown of genes on key pathways

## Conclusion and Future Perspective

The yin and yang of novel antibiotic discovery swings around from a serious need for antibiotics to eradicate drug resistant bacteria to antimicrobial agents. Ever increasing resistance despite the rigorous attempts for control use of antibiotics and sanitize hospitals routinely is the driver for the need of next generation antibiotics. These bacteria “superbugs” have escaped the clinical settings and attain the status of community acquired pathogens. Besides the upcoming promising antiinfective agents as discussed in this article earlier, there is the need to adopt alternative approaches for the discovery of next generation of antibiotics. The strategies discussed in this review are network pharmacology and functional genomics in complement with *in vivo* imaging platforms. The network pharmacology encompasses systems biology, network analysis, connectivity, redundancy, and pleiotropy to improve clinical efficacy and understanding side effects and toxicity. Functional genomics techniques are proved to be indispensible for *in vitro* target authentication and elucidating mechanism of action of novel antibacterial. In addition, the approach of functional genomics not only relies on target-based screening but also supports the traditional screening strategy by predicting novel mechanism for hits and natural products with potential antibacterial activity and unknown targets. Complementary to approach of drug discovery, *in vivo* imaging is an economical, user friendly and radioactive free tool that can extract quick semi-qualitative or quantitative information representing physiological milieu from the cellular to the computable form. Key for best realization of imaging and post-translational pathway modeling approaches into routine antibiotic discovery relies on its early integration with target specific drug development efforts. Validating efficiency and safety profiles of hit molecules in pathological models, before cost intensive medicinal approaches, offer a perfect solution for reducing the unsustainable expense. In summary, there is strong likelihood that the combination of both the traditional and genomics-based approaches, together with the live cell visualization techniques, will led to better strategies for the discovery of newer antibiotics to combat the emergence of bacterial resistance.

## Author contributions

SNK wrote review article. AUK guided and gave idea of writing.

## Conflict of Interest Statement

The authors declare that the research was conducted in the absence of any commercial or financial relationships that could be construed as a potential conflict of interest.
